# Forecasting shear stress parameters in rectangular channels using new soft computing methods

**DOI:** 10.1371/journal.pone.0229731

**Published:** 2020-04-09

**Authors:** Zohreh Sheikh Khozani, Saeid Sheikhi, Wan Hanna Melini Wan Mohtar, Amir Mosavi

**Affiliations:** 1 Institute of Structural Mechanics, Bauhaus Universität Weimar, Weimar, Germany; 2 Department of Civil Engineering, Faculty of Engineering & Built Environment, Universiti Kebangsaan Malaysia, Bangi, Selangor, Malaysia; 3 Department of Computer, Gorgan Branch, Islamic Azad University, Gorgan, Iran; 4 School of the Built Environment, Oxford Brookes University, Oxford, United Kingdom; 5 Department of Mathematics and Informatics, J. Selye University, Komarno, Slovakia; University of Glasgow, UNITED KINGDOM

## Abstract

Shear stress comprises basic information for predicting the average depth velocity and discharge in channels. With knowledge of the percentage of shear force carried by walls (%*SF*_*w*_) it is possible to more accurately estimate shear stress values. The %*SF*_*w*_, non-dimension wall shear stress ($\frac{{\bar{\tau }}_{w}}{\tau_{0}}$) and non-dimension bed shear stress ($\frac{{\bar{\tau }}_{b}}{\tau_{0}}$) in smooth rectangular channels were predicted by a three methods, the Bayesian Regularized Neural Network (BRNN), the Radial Basis Function (RBF), and the Modified Structure-Radial Basis Function (MS-RBF). For this aim, eight data series of research experimental results in smooth rectangular channels were used. The results of the new method of MS-RBF were compared with those of a simple RBF and BRNN methods and the best model was selected for modeling each predicted parameters. The MS-RBF model with *RMSE* of 3.073, 0.0366 and 0.0354 for %*SF*_*w*_, $\frac{{\bar{\tau }}_{w}}{\tau_{0}}$ and $\frac{{\bar{\tau }}_{b}}{\tau_{0}}$ respectively, demonstrated better performance than those of the RBF and BRNN models. The results of MS-RBF model were compared with three other proposed equations by researchers for trapezoidal channels and rectangular ducts. The results showed that the MS-RBF model performance in estimating %*SF*_*w*,_ $\frac{{\bar{\tau }}_{w}}{\tau_{0}}$ and $\frac{{\bar{\tau }}_{b}}{\tau_{0}}$ is superior than those of presented equations by researchers.

## Introduction

The determination of shear stress distribution is key in identifying the hydraulic induced sediment transport phenomena. Shear stress parameter ascribes to the fluid-sediment interaction particularly in the threshold of erosion and depositions, sediment incipient motion, and transport rates [1,2]. The boundary layer or near bed shear stress determines the mode of sediment transport either suspension or as bed load movement. Channel boundaries encompass of bed and wall, whereby the shear force acting can be determined based on the divisions of the weight of flowing water flowing onto the boundary [3]. Percentage of shear force %*SF*_*w*_ on each boundary is the ratio of (wall or bed) shear force to the shear force on the wetted perimeter [4]. Bed shear stress dominantly is responsible for the calculation of bedload transport and wall shear stress becomes effective in the discussion of channel migration and bank erosion.

Experimental, analytical and numerical procedures to determine the shear stress distribution in various shapes of channels have been exhaustively investigated [3–10]. Bed shear stress comprises of three terms, i.e. gravitational, secondary flows and interfacial shear stress. Early researchers considered negligibility in the last two terms (i.e. the secondary flows and interfacial shear stress) to simplify the flow conditions and only regarded the gravitational component. However, recent research reported that both secondary flows and interfacial stresses should be included to have an accurate representation of both bed and wall shear stress [11].

The complexity of natural flow behavior and interaction has been recently supervised using soft computing methods. These advanced artificial intelligence approaches proved to be successful in capturing the dynamicity of flow and able to predict hydraulics related output with high accuracy [12,13]. The use of the ability of Artificial Intelligent (AI) in data-driven modelling, percentage of shear force carried by flood plain walls in compound channels was successfully predicted [14]. Favourable prediction was also achieved for the estimation of shear stress distribution in circular channels using Extreme Learning Machine (ELM) model [15]. By assuming the dimensionless shear stress is a random variable, the entropy concepts of Renyi, Tsallis, and Shannon was able to minimise (if not eliminate) the uncertainty and accurately model the shear stress distribution along the channel boundaries [13].

The complexity of boundary shear stress limits available data through experimental and analytical work. Using the available data, the nonlinear modelling of Artificial Neural Network (ANN) is able to predict and forecasting the shearing behaviour at the boundary. The method allows for interpretation of boundary shear values without requiring explicit mathematical representations or further experimental work. The prediction can be made by a non-AI specialist as the application of ANN is relatively easy and the package is readily available within major software such as MATLAB.

Therefore, a new MS-RBF, simple RBF and BRNN model are utilized to estimate three parameters: percentage of shear force carried by walls, non-dimension wall shear stress and non-dimension bed shear stress in smooth rectangular channels. RBF boasts several advantages including faster training process, inherent approximation network, precise initial states and performs good with noisy data [16]. The three models are compared to identify the more robust model in predicting each parameter. Finally, the best model is compared with nonlinear regression equations used in trapezoidal channels and ducts for performance evaluation.

## Materials and methods

### Used data

**Fig 1:**
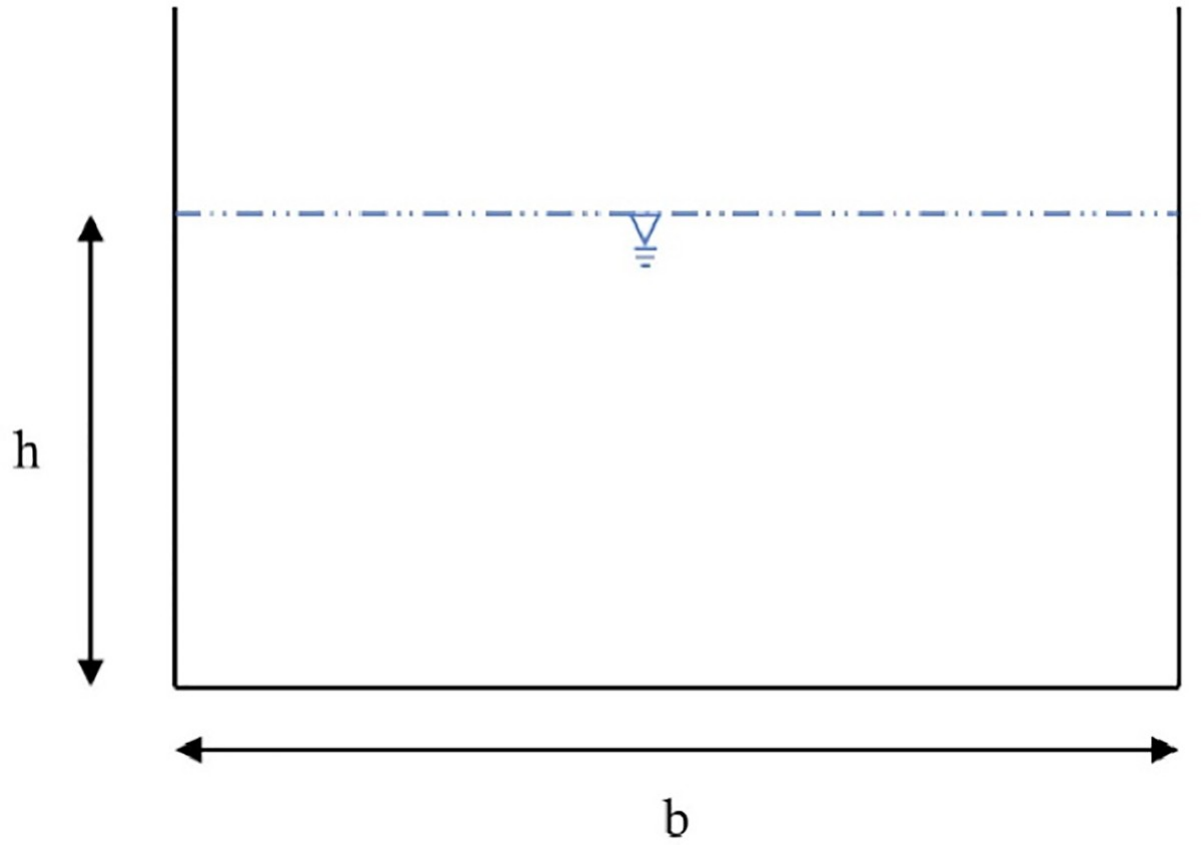
Rectangular channel cross section and notation.

Eight series of experimental data are used to predict the percentage of shear force carried by walls in smooth rectangular channels. These experiments introduce aspect ratio (*b*/*h*) (where *b* is the channel width, and *h* is the flow depth) as the most important parameter for shear stress values. Fig 1 demonstrates the rectangular channel cross section and its notation. The data employed in this study is obtained from [4,17–23]. Table 1 shows the range of data used.

**Table 1:** Ranges of the input and output parameters used for modelling.

Parameters References	*b*/*h*			*%SF*_*w*_			$\frac{{\bar{\tau }}_{w}}{\tau_{0}}$			$\frac{{\bar{\tau }}_{b}}{\tau_{0}}$		
	Min	Max	SD	Min	Max	SD	Min	Max	SD	Min	Max	SD
Cruff [17]	4.00	50.00	17.66	2.5	28.90	10.22	0.58	0.63	0.02	0.71	0.98	0.10
Ghosh and Roy [18]	1.91	3.12	0.72	36.4	76.20	14.83	0.43	0.57	0.05	0.24	0.64	0.15
Kartha and Leutheusser [23]	1.00	12.50	6.14	7.80	74.10	33.32	0.37	0.53	0.08	0.36	0.92	0.28
Myers [19]	1.56	19.12	6.16	6.90	56.50	20.01	0.44	0.67	0.08	0.44	0.93	0.20
Knight [4]	1.51	14.79	4.85	8.30	59.40	20.73	0.45	0.71	0.09	0.41	0.92	0.21
Noutsopoulos and Hadjipanos [42]	1.00	12.50	6.14	7.80	74.1	33.32	0.37	0.53	0.08	0.36	0.92	0.28
Knight et al. [21]	0.31	1.34	0.39	22.00	85.20	0.13	0.67	0.13	0.16	0.15	0.78	0.20
Seckin et al. [22]	6.59	14.80	2.69	11.83	18.44	2.84	0.54	0.61	0.03	0.82	0.87	0.02

Some nonlinear regression equations were produced by the above-mentioned researchers from experimental studies, all of which indicate that %*SF*_*w*_ is dependent on aspect ratio. They additionally presented equations for $\frac{{\bar{\tau }}_{w}}{\tau_{0}}$ and $\frac{{\bar{\tau }}_{b}}{\tau_{0}}$.

The equation proposed by [24] was obtained from experimental data for fully developed turbulent flow in a smooth rectangular duct with different depths as:

$$\%SF_{w}=\frac{103.23}{{\left(1+(\frac{2b}{3h})\right)}^{1.4128}}$$ (1)

$$\frac{{\bar{\tau }}_{w}}{\tau_{0}}=\left(\frac{\%SF_{w}}{100}\right)\left(\frac{b}{2h}\right)$$ (2)

$$\frac{{\bar{\tau }}_{b}}{\tau_{0}}=1-0.01\%SF_{w}$$ (3)

where *b* is the channel width, *h* is the water depth, *τ*_0_ = *ρgRS* (*ρ* is the fluid density, *S* is the bed slope of the channel, and *R* is the hydraulic radius), %*SF*_*w*_ is the percentage of shear force carried by walls, ${\bar{\tau }}_{w}$ and ${\bar{\tau }}_{b}$ are mean wall and bed shear stresses.

Rhodes and Knight [25] proposed the following equation for the percentage of shear force carried by walls in ducts:

$$\%SF_{w}=\frac{100}{1+{\left(\frac{1+1.345(h/b)}{1+1.345(b/h)}\right)}^{-1.057}}$$ (4)

Eqs (2) and (3) were proposed for non-dimension wall and bed shear stress respectively.

Using the concept of the relationship between *%SF*_*w*_ and wetted perimeter ratio (*P*_*b*_/*P*_*w*_), Flintham and Carling [26] presented a general equation for trapezoidal channels:

$$\%SF_{w}=e^{\alpha }$$ (5)

where *α* is a function of wetted perimeter ratio. By plotting log-log scales and assuming a simple relationship between %*SF*_*w*_ and *P*_*b*_/*P*_*w*_, the following equation is derived:

$$\log\left(\%SF_{w}\right)=C_{1}\;\log\left(\frac{P_{b}}{P_{w}}+C_{2}\right)+C_{3}$$ (6)

where *C*_*1*_, *C*_*2*_ and *C*_*3*_ are coefficients, and the limiting case is fixed by defining %*SF*_*w*_ = 100% for $\frac{P_{b}}{P_{w}}=0$ and then eliminating one constant. Therefore,

$$C_{3}=2-C_{1}\;\log\left(C_{2}\right)$$ (7)

Thus, the *α* parameter of Eq (5) is given by:

$$\alpha =-3.23\;\log\left(\frac{P_{b}}{P_{w}}+1\right)+4.6052$$ (8)

Eqs (2) and (3) were also proposed for non-dimension wall and bed shear stress. Since the results of numerous researchers show that the shear stress is dependent on aspect ratio (*b*/*h*), in the present study only this parameter was applied as input combination for all models.

### Bayesian regularized neural network

**Fig 2:**
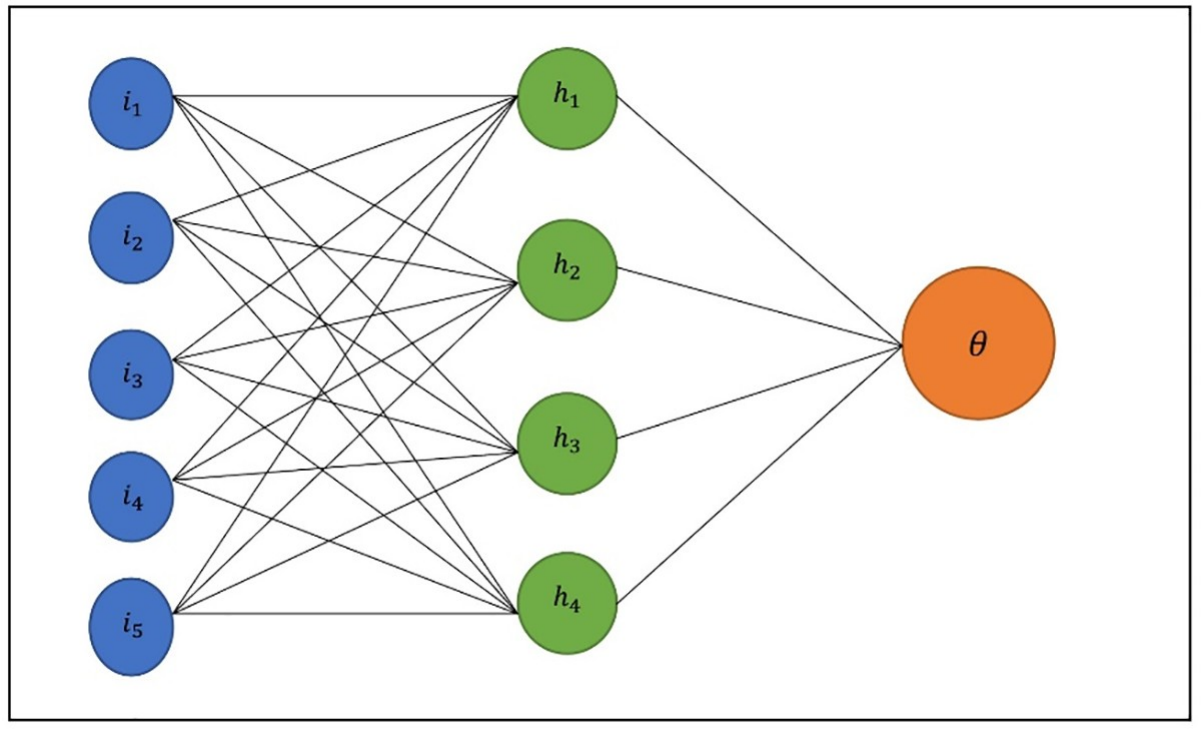
The structure of the two-layer neural network.

The Bayesian regularized neural network (BRNN) is one of the algorithms that has become popular nowadays for solving non-linear problems [27]. The Bayesian regularized neural network (BRNN), is one of the most efficient algorithms for solving regression problems. This algorithm implements the two-layer Bayesian regularized neural network, as introduced in [28] and [29]. The schematic structure of the two-layer neural network represented in Fig 2.

The input of neural network is set *X* = {*X*_1_.*X*_2_.….*X*_*n*_}. In order to provide a weighted linear combination, first input values are manipulated by sets of weights. This combination uses a nonlinear function to transform and create values of the state of neurons in the hidden layer. In general, this algorithm contains three layers of input, hidden and output. The hidden layer could consist of a different number of hidden neurons, while output layers include only one neuron and linear and sigmoid transfer functions are used to reach the neuron output.

**Fig 3:**
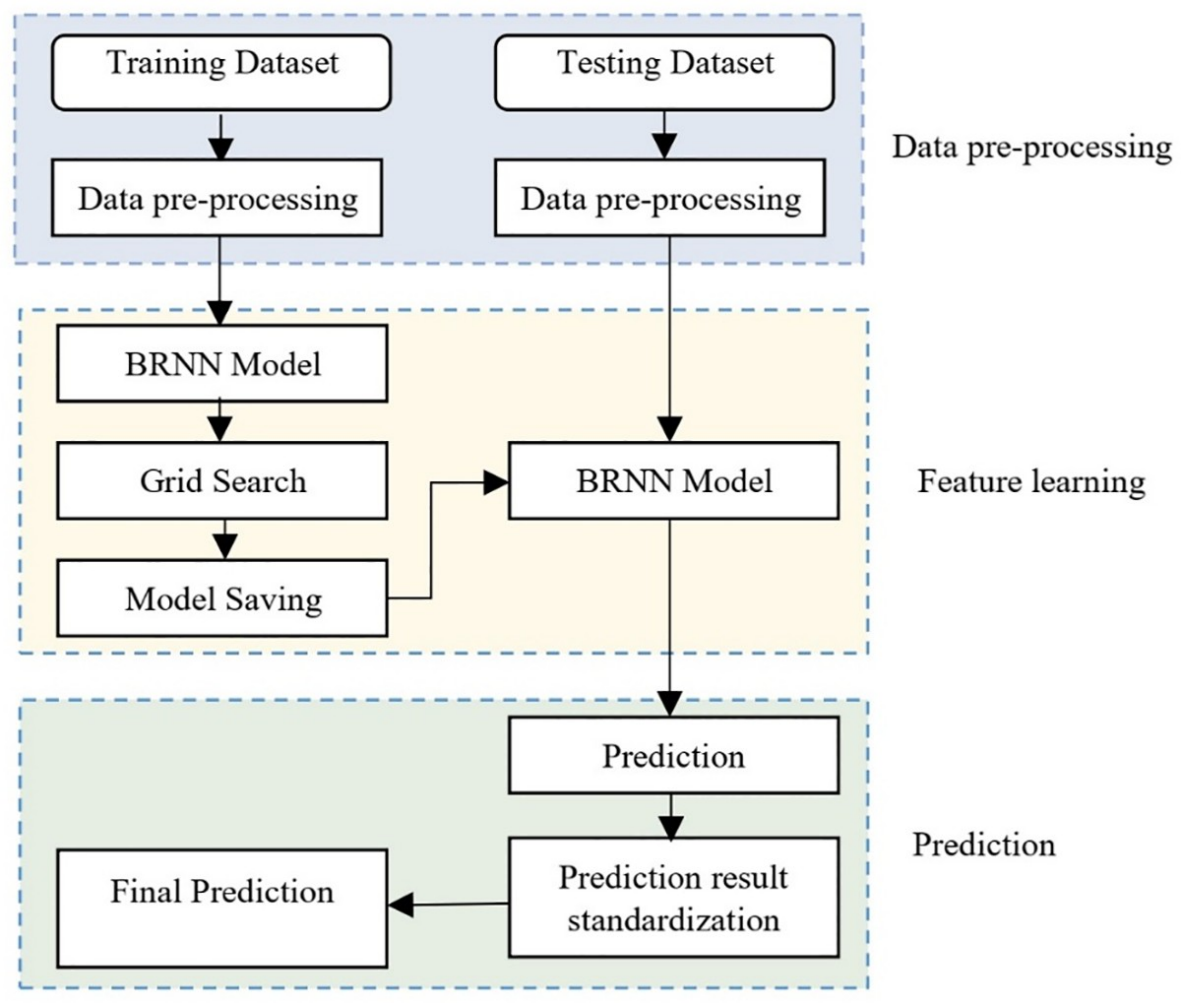
The BRNN structure.

The BRNN algorithm assigns initial weights to neurons using the Nguyen-Windrow initialization algorithm in order to nearly equally distribute the active regions of the layer’s neurons over the input space. This algorithm also optimizes result with the help of the Gauss-Newton method. Moreover, to improve the regression result of BRNN algorithm, we scaled input information for the training set to normalization information and rescaled the training data to be in the format of [0, 1]. This process is done once that the model is fitted. In the prediction phase, we used the testing set in the original scale; in this case, the algorithm rescaled testing information by employing normalization information that it acquired from the training set. It is then mapped back the output information to the original scale to represent results. Furthermore, we tuned the algorithm using a grid search method to find an optimal number of neurons with the highest performance for various train and test set of information. This process had a positive impact on train and prediction results and led the algorithm to achieve more accurate results on short process time. Fig 3 demonstrated the structure of BRNN algorithm.

### Radial basis function neural network

RBF neural networks [30,31] are among the most common artificial intelligence methods utilized for simulating various hydraulic engineering problems. An RBF consists of three different layers: the input, hidden and output layers layer. The input layer introduces the input variables to the model and transfers them to the hidden layer. The hidden layer reduces the experimental dimensionality through a non-linear projection of the input variables. The projection is done by some functions known as “radial basis functions”. The radial basis function, φ(x,c), is a real function value which depends on the original distance [32], where x and c are the input variable and center of the function, respectively. Thus, a radial basis function value changes only with a change in radial distance and is defined as follows:

r=‖x–c‖ (9)

where r denotes to the Euclidean norm, and x is an input variable, and finally, c refers a centroid.

By running the RBF neural network, an N-dimensional linear function is constructed as:

$$\left\{\phi (||x-x_{i}||)|i=1,2,\ldots ,N\right\}$$ (10)

Following the hidden layer, a linear regression is done in the output layer. Therefore, the RBF neural network results are determined using the weighted summation of the hidden layer neurons. The coefficients of this weighted summation are determined using the least squared method, and the results are presented as follows:

$$f(x)=\sum_{i=1}^{N}c_{i}\phi (||x-x_{i}||)$$ (11)

**Fig 4:**
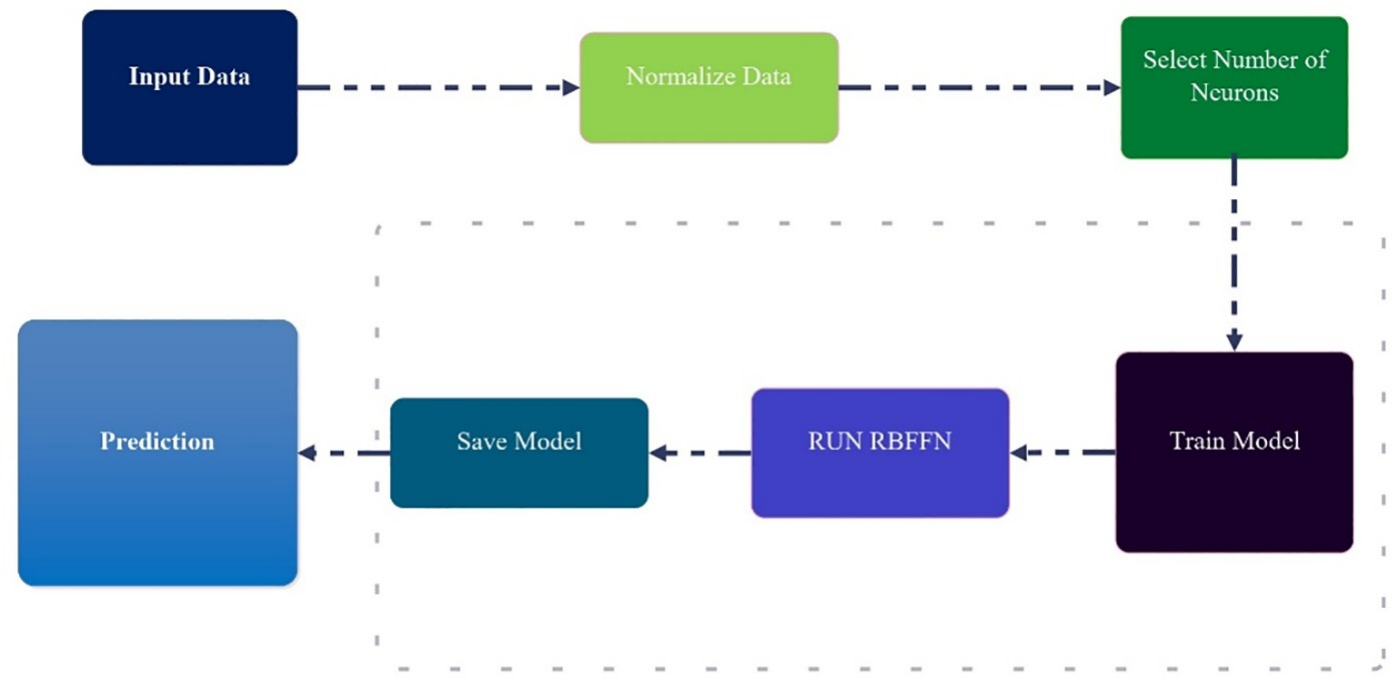
The diagram of the RBF model.

The number of input layer neurons is equal to the number of input variables, and the number of output layer neurons and output variables is the same. However, there is no particular rule to determine the number of hidden layer neurons. In the present study, trial and error is done in order to determine the number of hidden layer neurons [33,34]. The diagram of the RBF illustrated in Fig 4.

### Hybrid decision tree-based neural networks

The novel hybrid MS-RBF method is introduced in this section. A DT classification problem [35] comprises a number of variables including class variables (Y) and input variables (X). Y takes amounts between 1 and k, where k is the number of classes, considered for the problem. For each sample set, X is equal to X1 to Xp, where p is the number of input variables. Predicting the class of a new sample (Y) by using the input variables (X) is the goal of the DT algorithm. A DT algorithm applies recursive partitioning in order to classify problems. This method has the advantage of no limitation on the number of input variables. The DT algorithm is discussed by [35] in detail. The power allocation of the RBF method to the dataset is optimized by the hybrid MS-RBF method. In this hybrid approach, despite assigning the entire power of an RBF neural network to the whole dataset, the RBF and the dataset are partitioned into some parts. The procedure of the MS-RBF method is as follows: (1) Dataset division: In the MS-RBF method, first the whole dataset is divided into three parts according to the amounts of output variables. Therefore, the entire dataset is partitioned into three types of samples, with low, medium, and high amounts of output variables. It is worth nothing here that the percent of each sample type (low output, medium output, and high output samples) is determined by MS-RBF via trial and error.

**Fig 5:**
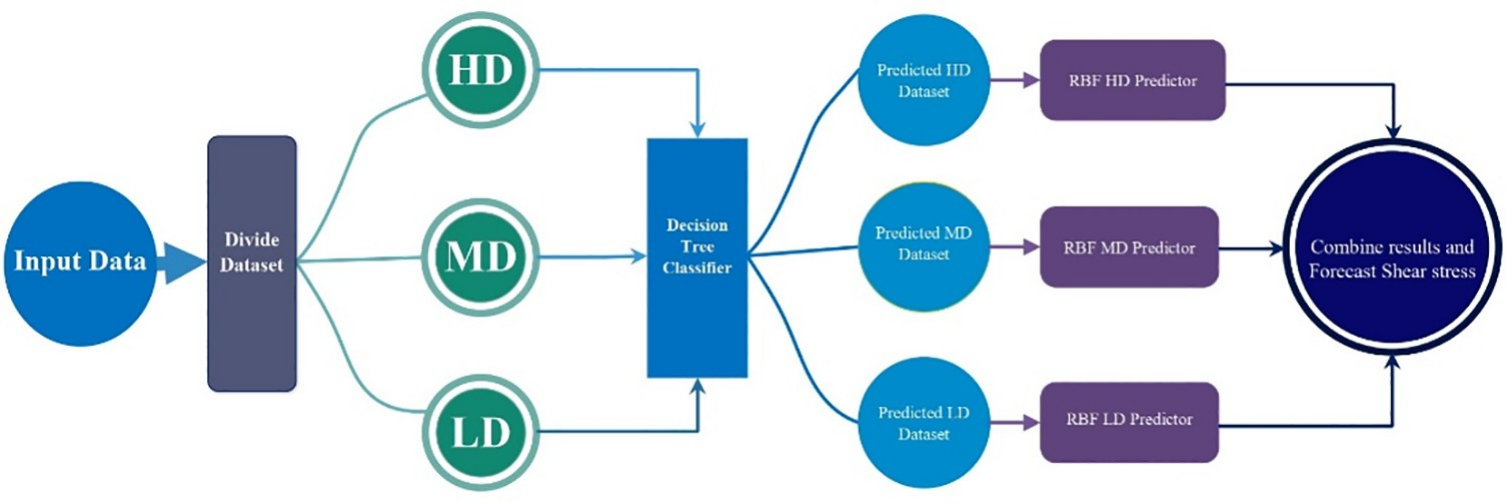
The structure of MS-RBF model.

(2) DT training: After dividing the dataset, the DT is trained with the input variables of the problem as X variables, and “Low,” “Medium,” and “High” as the Y variables of the classification problem. An important element in DT training is classification precision. With high precision, misclassified samples can be arranged; however, the DT structure becomes very complex. On the other hand, weak classification would lead to a simple and easy-to-use DT structure, but the number of misclassified samples would be high. In the MS-RBF method, the trial and error is utilized to find the optimum precision of DT classification.

(3) RBF partitioning: In this step, the RBF neural network is split into three smaller RBF models. In this study, the maximum allowable number of hidden layer neurons of the main RBF is 12. Therefore, the sum of the maximum allowable number of hidden layer neurons of the smaller RBF is 12.

(4) Finding the RBF optimum structure: Now, trial and error method is utilized to determine the number of hidden layer neurons in the main RBF model and three smaller RBF models (by considering the maximum allowable number of hidden layer neurons from the previous step).

(5) Result combination: In order to compare the results of the main, simple RBF model with the hybrid MS-RBF, the results of the three smaller RBFs are combined. In this paper, the MS-RBF model is utilized to simulate the percentage of shear force carried by walls, non-dimension wall shear stress and non-dimension bed shear stress in smooth rectangular channels. This modeling procedure of MS-RBF technique is shown in Fig 5. In this figure five steps can be seen that each step is as:

Step 1: Input dataset into the MS-RBF model.
Step 2: Divide the entire dataset into three types of samples, with low (LD), medium (MD), and high (HD) amounts of output variables.
Step 3: Generate the classifications of the three groups HD, MD, and LD using the Decision Tree classifier.
Step 4: Input the three predicted datasets of HD, MD, and LD to the RBF-HD, RBF-MD, and RBF-LD predictor models.
Step 5: Combine all results of separated RBF neural network models and Forecast Shear stress.

## Results

### Performance evaluation

The performances of models were evaluated using some global statistics: These applied criteria are as Correlation Coefficient (R^2^), Root Mean Square Errors (RMSE), Mean Absolute Error (MAE), Nash-Sutcliffe Efficiency (NSE), and Percentage of BIAS (PBIAS) that defined as:

$$R^{2}={\left(\frac{\sum_{i=1}^{n}\left(x_{im}-{\bar{x}}_{im})(x_{ip}-{\bar{x}}_{ip}\right)}{\sqrt{\sum_{i=1}^{n}{\left(x_{im}-{\bar{x}}_{im}\right)}^{2}\times {\sum_{i=1}^{n}\left(x_{ip}-{\bar{x}}_{ip}\right)}^{2}}}\right)}^{2}$$ (12)

$$RMSE=\sqrt{\frac{\sum_{i=1}^{n}{\left(x_{ip}-x_{im}\right)}^{2}}{n}}$$ (13)

$$MAE=\frac{1}{n}\sum_{i=1}^{n}\left|x_{ip}-x_{im}\right|$$ (14)

$$NSE=1-\frac{\sum_{i=1}^{n}{\left(x_{im}-x_{ip}\right)}^{2}}{\sum_{i=1}^{n}{\left(x_{im}-{\bar{x}}_{im}\right)}^{2}}$$ (15)

$$PBIAS=100\times \left[\frac{\sum_{i=1}^{n}\left(x_{im}-x_{ip}\right)}{\sum_{i=1}^{n}x_{ip}}\right]$$ (16)

where, *x*_*ip*_ denotes the value of predicted parameter by the model, *x*_*im*_ is the measured values in the experiments, and ${\bar{x}}_{im}$ and ${\bar{x}}_{ip}$ are the mean of measured and predicted values respectively.

### The percentage of shear force carried by walls modeling

**Fig 6:**
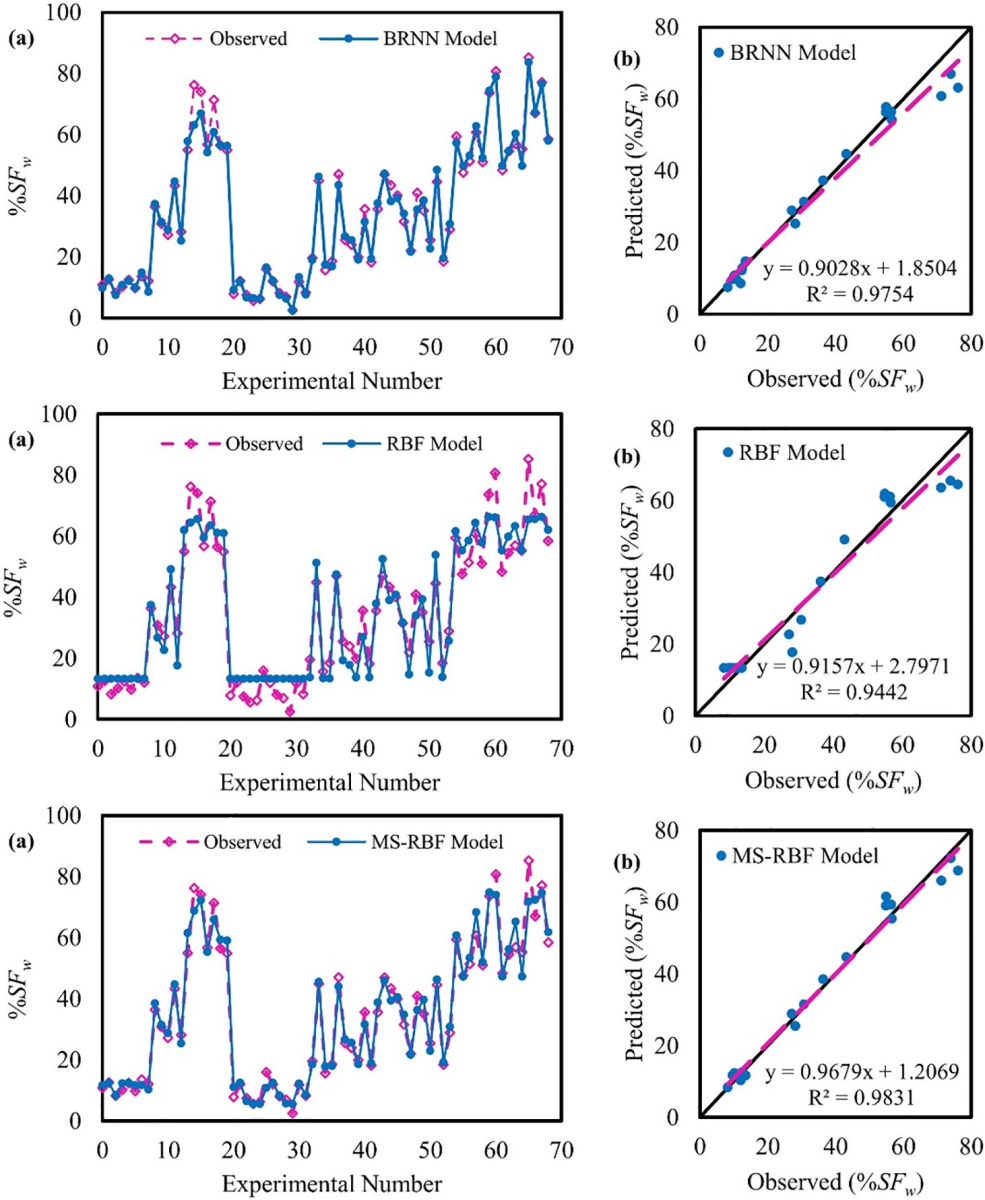
Observed and estimated percentage of shear force values: (a) for the entire dataset and (b) for the testing dataset.

The BRNN, RBF and MS-RBF estimates for the entire dataset and testing period were compared with observed wall shear force and are illustrated Fig 6. The MS-RBF with *R*^2^ of 0.98 performed better than simple RBF model with *R*^2^ of 0.94, and the results of MS-RBF model are close to results of BRNN model with *R*^2^ of 0.97. It is clear from the fit line equations (assuming the equation is *y* = *a*_0_*x* + *a*_1_) that the *a*_0_ and *a*_1_ coefficients for MS-RBF are closer to 1 and 0 respectively, than those of the simple RBF model and BRNN model. This confirms the statistical results which presented in Table 2. According to the results of Fig 6A, the MS-RBF model can estimate results closer to experimental data than those of the simple RBF and BRNN methods, meaning that the MS-RBF method produces much better estimations of %*SF*_*w*_.

**Table 2:** Performance evaluation of the BRNN, RBF and MS-RBF models for both test and train stage for modeling the shear force carried by walls.

Models		RMSE	MAE	NSE	PBIAS
BRNN	Test	4.35	2.63	0.97	-3.40
	Train	2.15	1.63	0.99	0.38
RBF	Test	5.46	4.59	0.94	6.01
	Train	6.69	5.61	0.91	16.97
MS-RBF	Test	3.07	2.34	0.98	1.47
	Train	3.66	2.53	0.97	2.57

Table 2 provides the statistical parameter values for predicting %*SF*_*w*_ by BRNN, RBF, and MS-RBF models for both the training and testing stages. As expected, the improved MS-RBF method with lower statistical parameter values exhibits superior performance over the simple RBF and BRNN methods. As seen in Table 2, the statistical values of the MS-RBF model are close to each other for testing and training, signifying that the model has no overturning in the modeling process of shear force carried by walls. According to the results of Table 2, the MS-RBF model with RMSE of 3.07 performs better than the simple RBF model with RMSE of 5.46 and BRNN model with RMSE of 4.35, respectively, for the testing dataset.

### Non-dimension wall shear stress modeling

**Fig 7:**
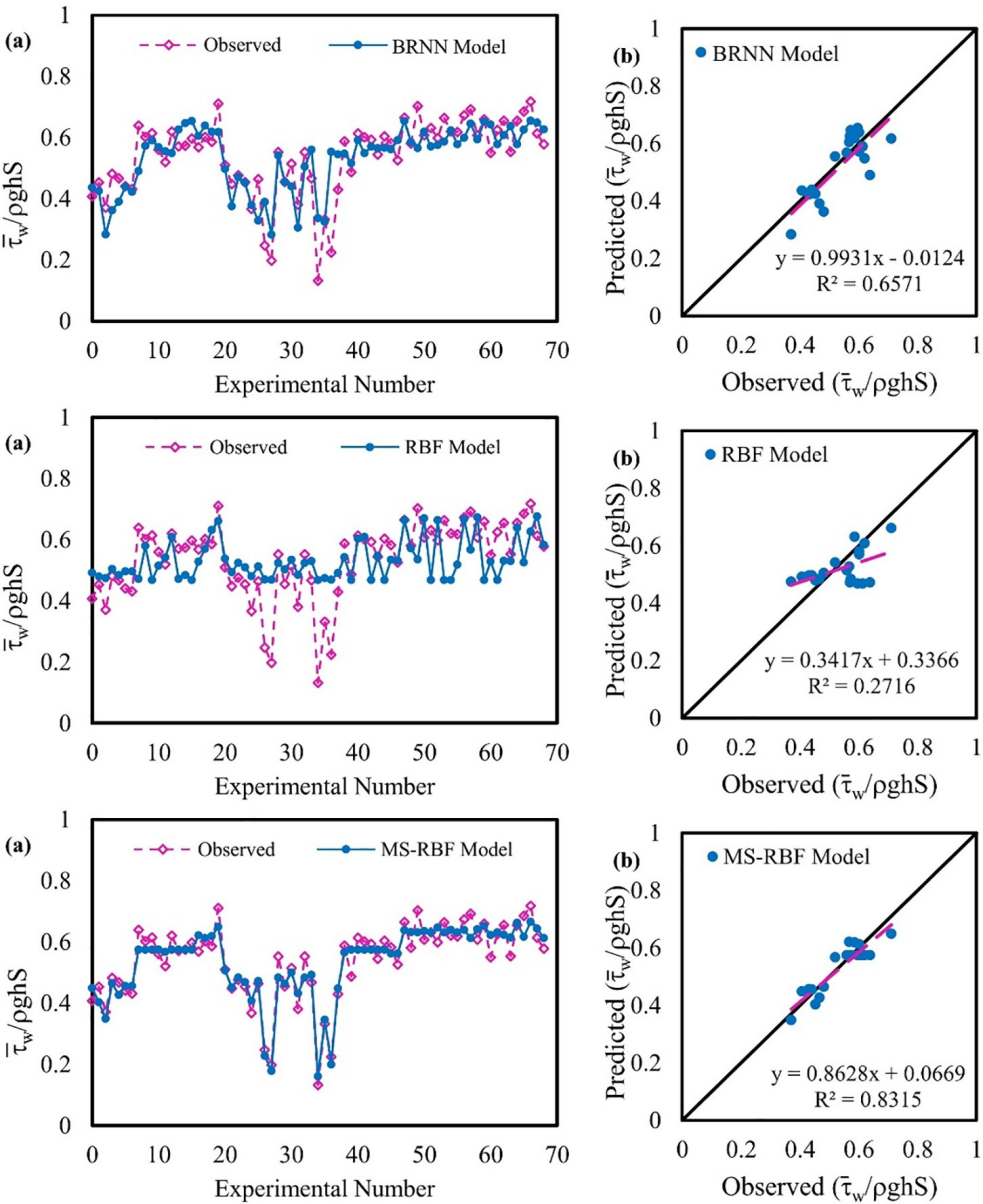
Observed and estimated non-dimension wall shear stress values: (a) for the entire dataset and (b) for the test dataset.

Fig 7 shows the observed and predicted values of $\frac{{\bar{\tau }}_{w}}{\tau_{0}}$ for all datasets and for the test dataset as a scatter plot for the BRNN, RBF and MS-RBF models. According to the figure, the MS-RBF model approximates the observed data better than both RBF and BRNN models. The MS-RBF model has an *R*^2^ coefficient of 0.8315, which is much higher than 0.2716 obtained with the simple RBF model. In the scatter plot, the trend line of values predicted by the MS-RBF model is much closer to the fitted line than the RBF model, indicating the greater performance of the improved RBF model than the simple RBF model. The results of BRNN model for estimating $\frac{{\bar{\tau }}_{w}}{\tau_{0}}$ is much better than simple RBF model for testing stage and whole dataset but the accuracy of BRNN in estimating $\frac{{\bar{\tau }}_{w}}{\tau_{0}}$ is lower than MS-RBF model. The MS-RBF model has better function than the BRNN and RBF model with respect to the fit line equation, since the *a*_0_ and *a*_1_ coefficients of the MS-RBF model are respectively closer to 1 and 0 than those of the BRNN and RBF models.

Non-dimension wall shear stress ($\frac{{\bar{\tau }}_{w}}{\tau_{0}}$) was simulated using BRNN, RBF and MS-RBF models. The statistical parameter simulation results for the testing and training stages are shown in Table 3. Clearly, the values of these parameters decrease as the RBF model improves, with RMSE of 0.04 for the MS-RBF model with the testing dataset compared with the RBF model with RMSE of 0.08. The results of BRNN predictions are close to the results of simple RBF model with RMSE of 0.07. For the MS-RBF model, all statistical parameters for the training dataset are close to the statistical parameters for testing stage, shows the superior performance of the MS-RBF model in estimating the $\frac{{\bar{\tau }}_{w}}{\tau_{0}}$. The statistical result values of the RBF model are increased in training stage in compare with the testing stage. Overall, the MS-RBF model with lower statistical parameter values (MAE of 0.03) shows better performance than the simple RBF model (MAE of 0.08) in predicting $\frac{{\bar{\tau }}_{w}}{\tau_{0}}$.

**Table 3:** Performance evaluation of the BRNN, RBF and MS-RBF models for both test and train stage for modeling the dimensionless wall shear stress.

Models		RMSE	MAE	NSE	PBIAS
BRNN	Test	0.07	0.05	0.36	-3.70
	Train	0.08	0.06	0.63	5.68
RBF	Test	0.08	0.07	-0.02	-1.76
	Train	0.12	0.09	0.24	10.12
MS-RBF	Test	0.04	0.03	0.81	-1.18
	Train	0.04	0.03	0.92	0.54

### Non-dimension bed shear stress modeling

**Fig 8:**
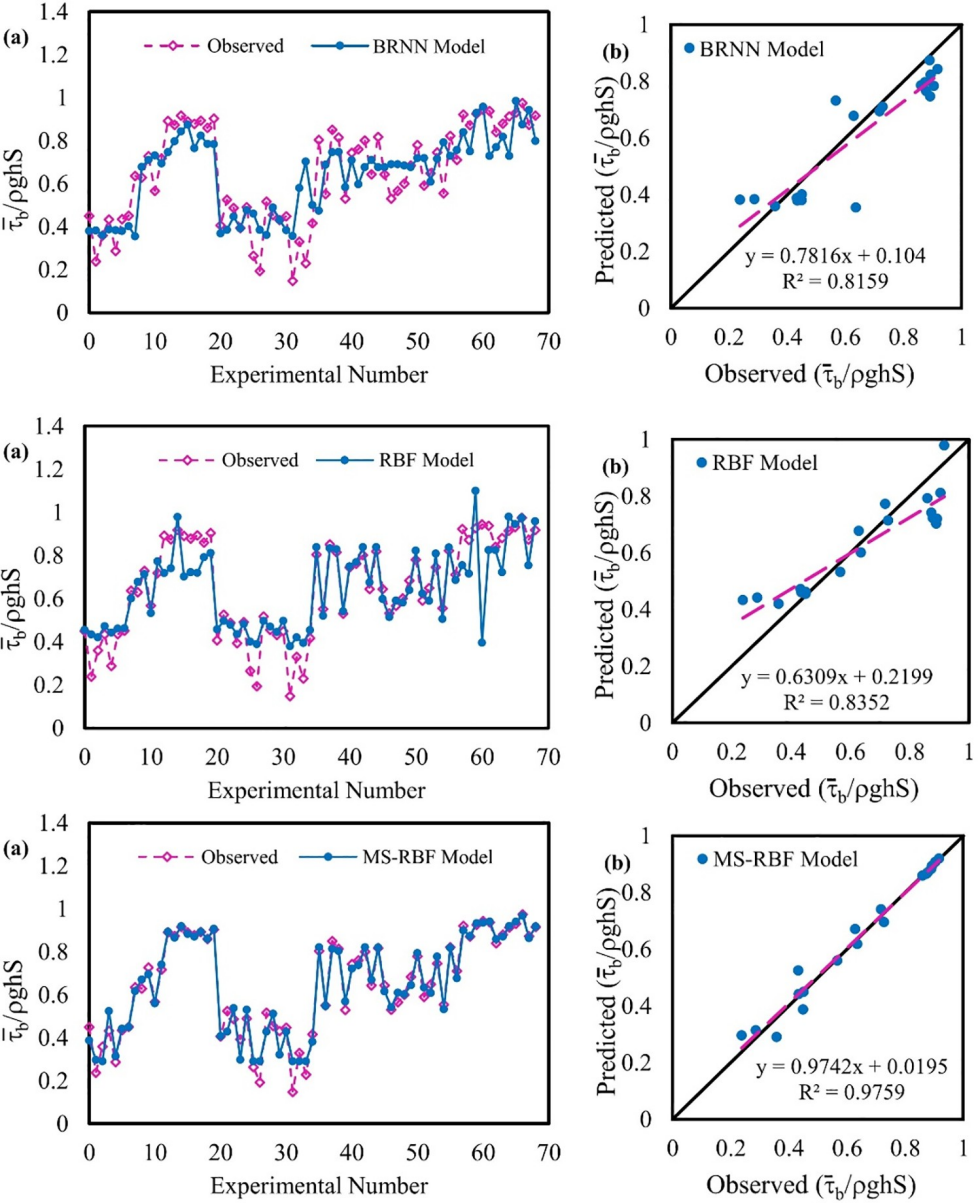
Observed and estimated non-dimension bed shear stress values: (a) for the entire dataset and (b) for the test dataset.

The BRNN, RBF and improved MS-RBF models were also used to model non-dimension bed shear stress. The results of all three models for estimating mean bed shear stress are compared in Fig 8. Evidently, the results of the MS-RBF model in all datasets are closer than those of the BRNN and RBF models to experimental data. Additionally, according to the scatter plot for the test dataset, the MS-RBF model with *R*^2^ of 0.9759 performs better than the BRNN and RBF models with *R*^2^ of 0.8159 and 0.8352 respectively. The *a*_1_ and *a*_2_ fit line equation coefficients of the MS-RBF model (0.9742 and 0.0195) respectively closer to 1 and 0 respectively, than those of the BRNN (0.7616 and 0.104) and RBF models (0.6309 and 0.2199).

Table 4 presents the RMSE, MAE, NSE and PBIAS values of the applied models for both training and testing stages. According to the results of Table 4 the BRNN model demonstrated the least accurate results for estimating dimensionless bed shear stress with the highest values of error for both training and testing stages. All statistical parameters values of the RBF model for the testing and training datasets are much bigger than those of the MS-RBF model. According to the results in Table 4, it can be deducted that the improved MS-RBF model performs much better than the simple RBF and BRNN models. The MS-RBF model enhancement has been so successful because for the test dataset the MS-RBF model with RMSE of 0.04 showed better function than the RBF model with RMSE of 0.10. Moreover, the statistical parameters values for the testing dataset are close to the training dataset, which suggests that the models performed well during modeling.

**Table 4:** Performance evaluation of the BRNN, RBF and MS-RBF models for both test and train stage for modeling the dimensionless bed shear stress.

Models		RMSE	MAE	NSE	PBIAS
BRNN	Test	0.11	0.08	0.77	-0.87
	Train	0.14	0.11	0.60	9.85
RBF	Test	0.10	0.08	0.78	6.24
	Train	0.11	0.07	0.74	6.83
MS-RBF	Test	0.04	0.03	0.97	1.43
	Train	0.05	0.03	0.96	2.27

### Comparison of MS-RBF model results and other equations for %*SF*_*w*_

**Fig 9:**
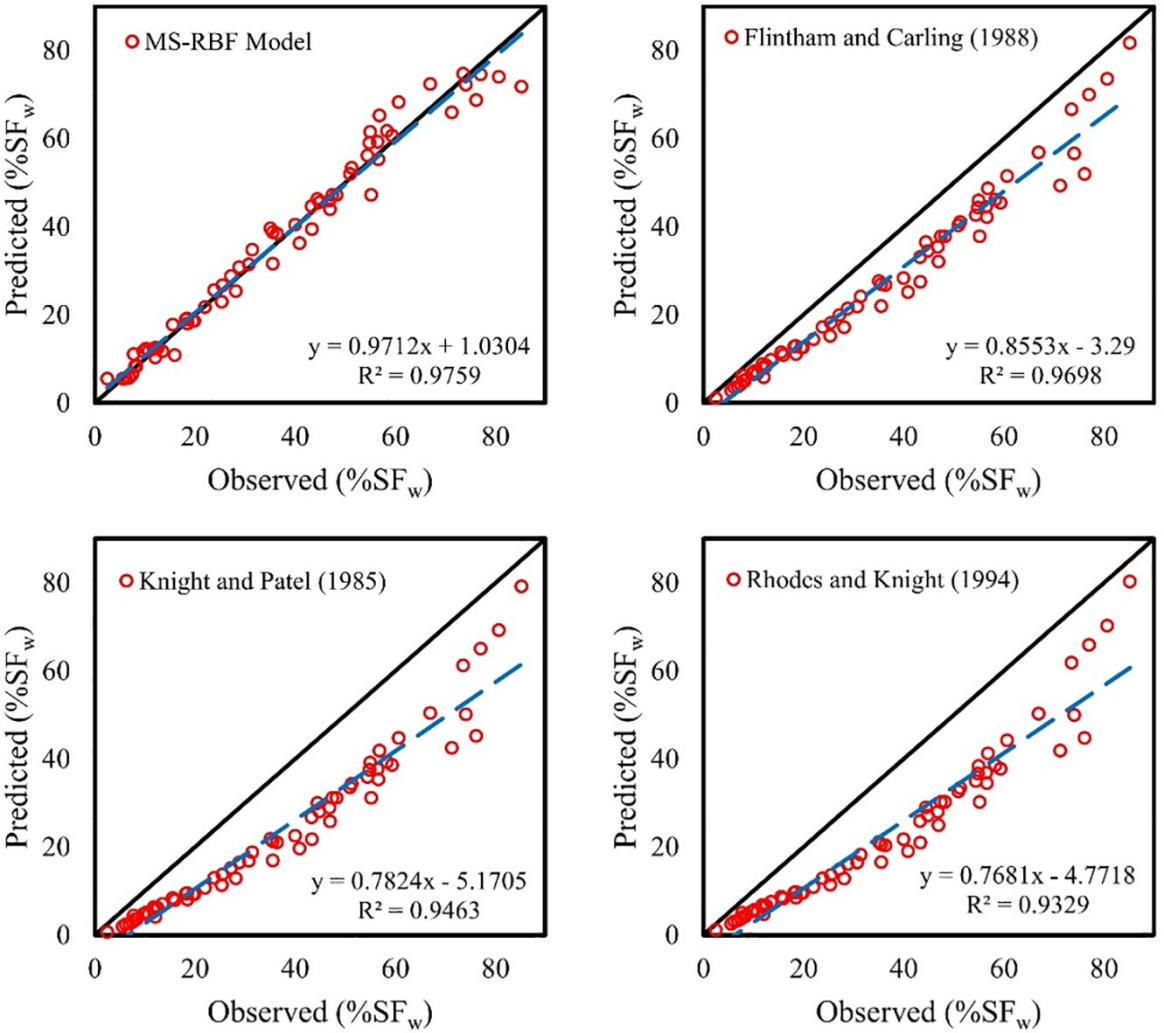
Scatter plot of comparison between the best model and other experimental equations for percentage of shear force.

Since the MS-RBF model demonstrated more appropriate results with the lowest statistical values in estimating %*SF*_*w*_, MS-RBF was selected as the best model and was compared with Eqs (1), (4) and (5). The results of this comparison are illustrated in Fig 9. The equation offered by [26] [Eq (5)] predicted underestimated values for %*SF*_*w*_ and so did the other two proposed equations for ducts [Eqs (1) and (4)]_._ The MS-RBF model with *R*^2^ of 0.9759 showed the best performance over Eqs (5), (1) and (3) with *R*^2^ of 0.9698, 0.9463 and 0.9329 respectively. The trend line of the MS-RBF model results is very close to the exact line, while other nonlinear regression equations’ trend lines are farther from the fitted line, which indicates the numerical model can predict more accurate %*SF*_*w*_ values than other equation. As seen from the fit line equations in the scatterplots, the *a*_0_ and *a*_1_ coefficients for the MS-RBF model are 0.9712 and 1.0304 respectively and are closer to 1 and 0 than those of other equations. According to the results of the comparison equations, the equation for trapezoidal channels [Eq (5)] exhibited better performance than duct equations. Generally, the MS-RBF model has a better function and high performance in predicting %*SF*_*w*_.

### Comparison of MS-RBF model results and other equations for $\frac{{\bar{\tau }}_{w}}{\tau_{0}}$

**Fig 10:**
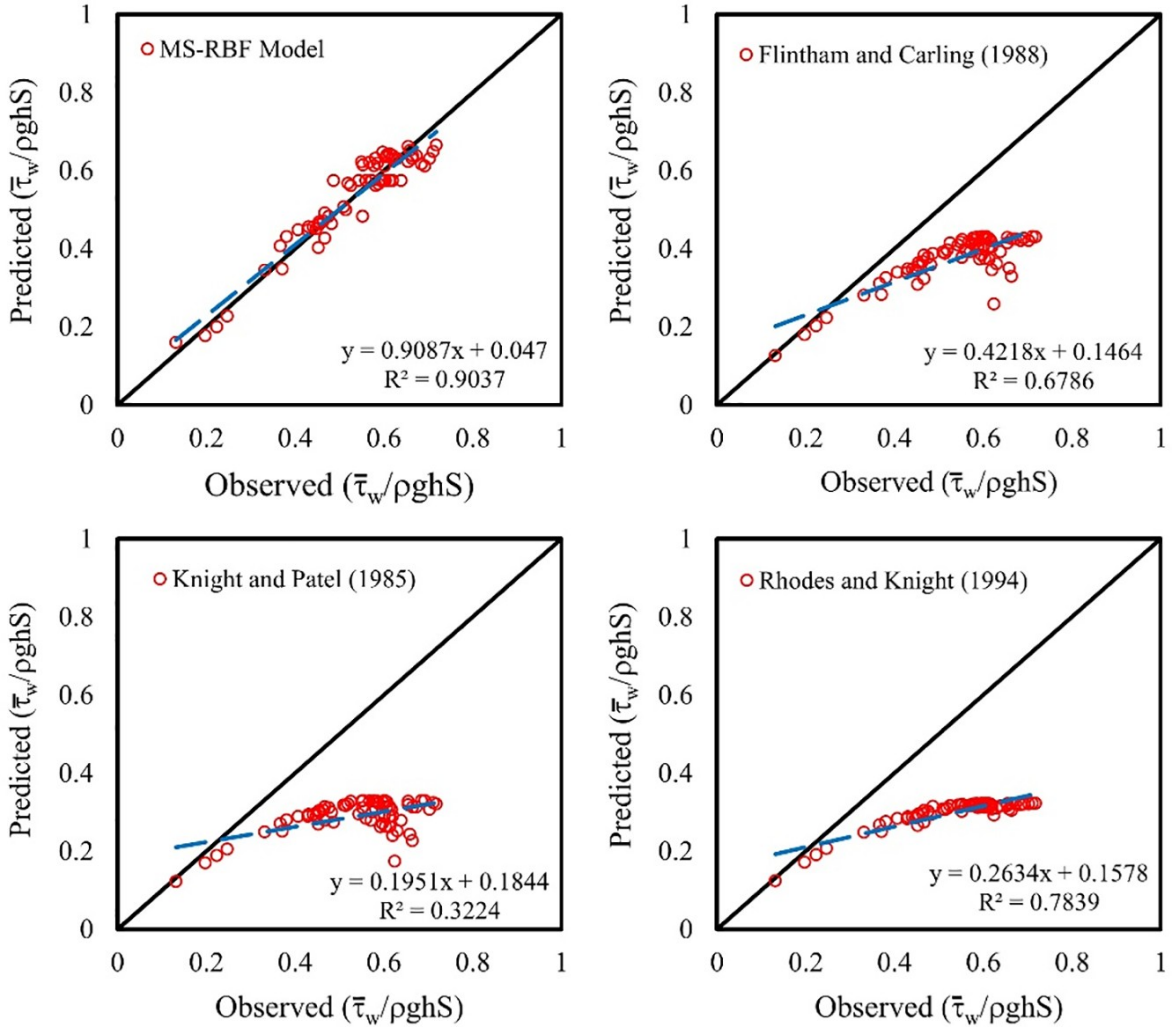
Scatter plot of comparison between the best model and other experimental equations for non-dimension wall shear stress.

The non-dimension wall shear stress results of the MS-RBF model and other nonlinear regression equations are represented in Fig 10 in the form of a scatterplot. This figure indicates the greater closeness of the MS-RBF model’s prediction of $\frac{{\bar{\tau }}_{w}}{\tau_{0}}$ with the target predictions than other equations. All three relations for trapezoidal channels and rectangular ducts showed underestimated values for $\frac{{\bar{\tau }}_{w}}{\tau_{0}}$, while the proposed model with *R*^2^ of 0.9037 showed high performance in predicting values for $\frac{{\bar{\tau }}_{w}}{\tau_{0}}$. The equations presented for rectangular ducts approximately estimate constant values for flow depth ranging from 0.4 to 0.75 and their trend lines are straight, meaning their results are not sufficiently accurate in predicting the target parameter. The trend lines of the equations from other studies indicate the poor function of the equations in estimating non-dimension wall shear stress; the *a*_0_ coefficients are 0.4218, 0.1951 and 0.2634 for the relations of Flintham and Carling [26], Knight and Patel [24] and Rhodes and Knight [25] respectively. These values are not close to 1, therefore the MS-RBF model with the highest *R*^2^ values performs better in estimating $\frac{{\bar{\tau }}_{w}}{\tau_{0}}$ than other equations.

### Comparison of MS-RBF model results and other equations for $\frac{{\bar{\tau }}_{b}}{\tau_{0}}$

**Fig 11:**
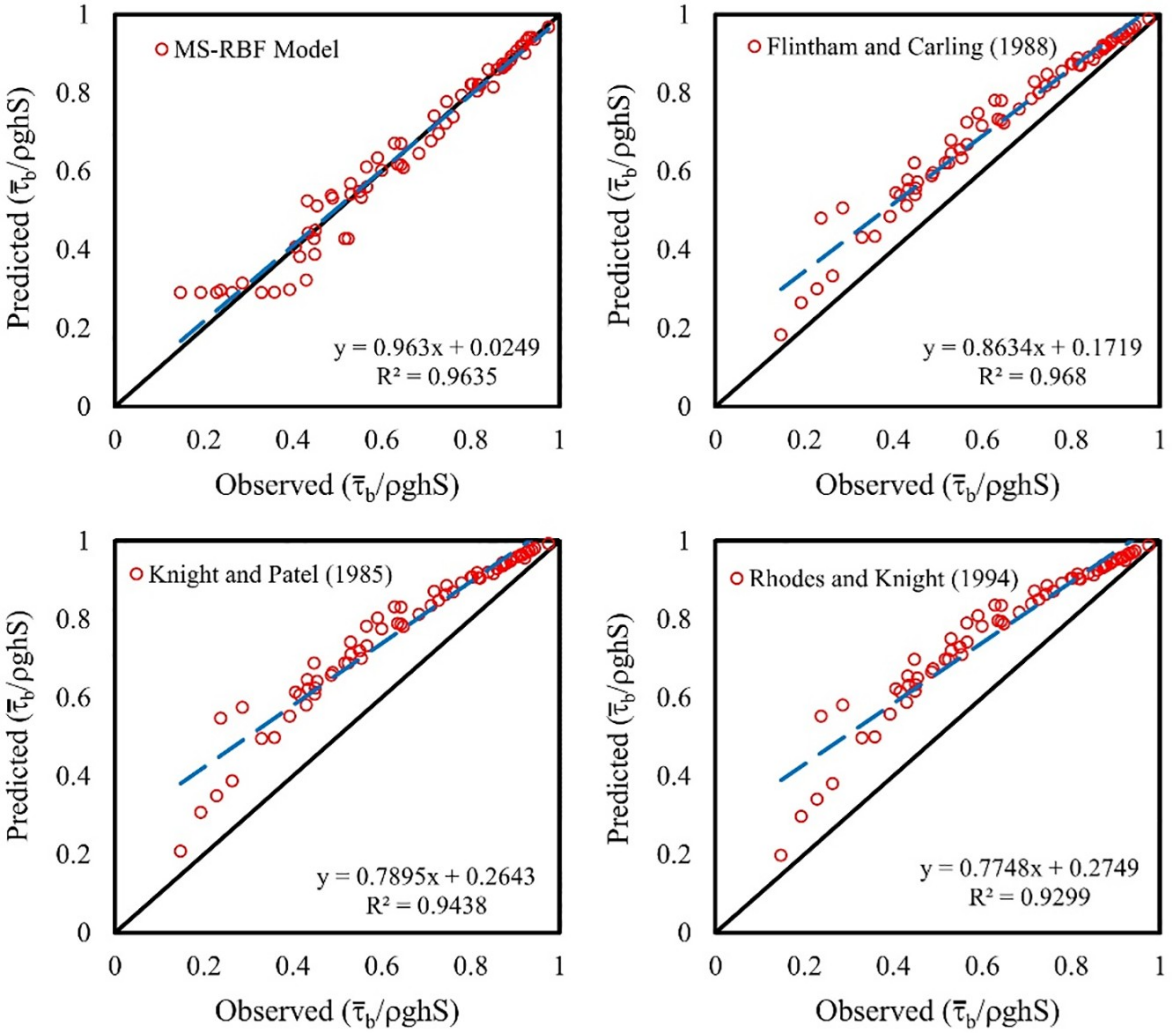
Scatter plot of comparison between the best model and other experimental equations for non-dimension bed shear stress.

The MS-RBF model results are compared with those of Eq (3) in terms of computation with different relations of percentage of shear force carried by walls. Fig 11 presents the results of this comparison. The MS-RBF model with results closer to experimental data performs the best among all methods. The trend line of the MS-RBF model matches the exact line with *R*^2^ of 0.9635, rather than equations for trapezoidal channels and rectangular ducts with *R*^2^ of 0.9680, 0.9438 and 0.9299 respectively. The equations proposed by other researchers predict overestimated values for $\frac{{\bar{\tau }}_{b}}{\tau_{0}}$, but the equation for trapezoidal channels performs better than the relations for rectangular ducts. The non-dimension bed shear stress values estimated by other relations show higher accuracy than equations for non-dimension wall shear stress, but the MS-RBF model performs the best among all equations.

## Discussion

The shear stress is one of the vital components in hydraulic of channels. The prediction of accurate values of shear stress due to designing more stable channels [13]. For prediction of shear stress distribution around wetted perimeter by using some analytical methods the mean wall and bed shear stresses also the percentage of shear force carried walls is needed [36–38]. Therefore, the more accurate results of these three parameters more accurate results of shear stress will be obtained. In this study three different methods are applied to estimate %*SF*_*w*_, dimensionless mean wall and bed shear stresses. The most important issue in this study is applying simple RBF model and modified structure of RBF and investigation of accuracy of MS-RBF model in estimation. Many researchers used simple models and then hybrid one to improve the results of predictions [39–41]. Sheikh Khozani et al. [40] applied some tree-based models to forecast apparent shear stress in compound channels. They investigated the best input combination which estimate more accurate results. In this study because we only have an input parameter as y/P then the structure of models is simpler than models with more input variables and no need to investigate the best input combination. In the study of Sheikh Khozani et al. [40] the hybrid model Bagging-M5P performed better than other simple models. In present study the MS-RBF model proved higher prediction power of hybrid algorithms in compare with simple RBF and BRNN models.

## Conclusion

Three shear stress parameters (%*SF*_*w*_, $\frac{{\bar{\tau }}_{w}}{\tau_{0}}$ and $\frac{{\bar{\tau }}_{b}}{\tau_{0}}$) were modeled with the BRNN, simple RBF and MS-RBF models and the most appropriate model for predicting these parameters was selected. Also, the estimated values by the best model are compared with three different nonlinear equations proposed by other researchers. The obtained results are as:

1. The MS-RBF model with RMSE of 3.073, 0.0366 and 0.0354 for predicting %*SF*_*w*_, $\frac{{\bar{\tau }}_{w}}{\tau_{0}}$ and $\frac{{\bar{\tau }}_{b}}{\tau_{0}}$ respectively for the test dataset, displayed better function compared with the BRNN and RBF models.
2. The MS-RBF model with *R*^2^ of 0.9757 showed higher ability in estimating % *SF*_*w*_ than other equations with *R*^2^ of 0.9698, 0.9463 and 0.9329 presented by Flintham and Carling [26], Knight and Patel [24] and Rhodes and Knight [25] respectively.
3. All nonlinear equations computed underestimated values for $\frac{{\bar{\tau }}_{w}}{\tau_{0}}$ while the MS-RBF model demonstrated the best performance with *R*^2^ of 0.9037.
4. In the comparison between the MS-RBF model’s results with other equations in estimating $\frac{{\bar{\tau }}_{b}}{\tau_{0}}$, the trend line of the MS-RBF model was closer to the fitted line with *R*^2^ of 0.9635 and the other equations predicted overestimated values for this parameter.
